# Supplementary figures and images for: Chemical classification program synthesis using generative artificial intelligence
Source: J Cheminform. 2025 Oct 1;17:152. doi: 10.1186/s13321-025-01092-3 (PMC12490122; doi:10.1186/s13321-025-01092-3)

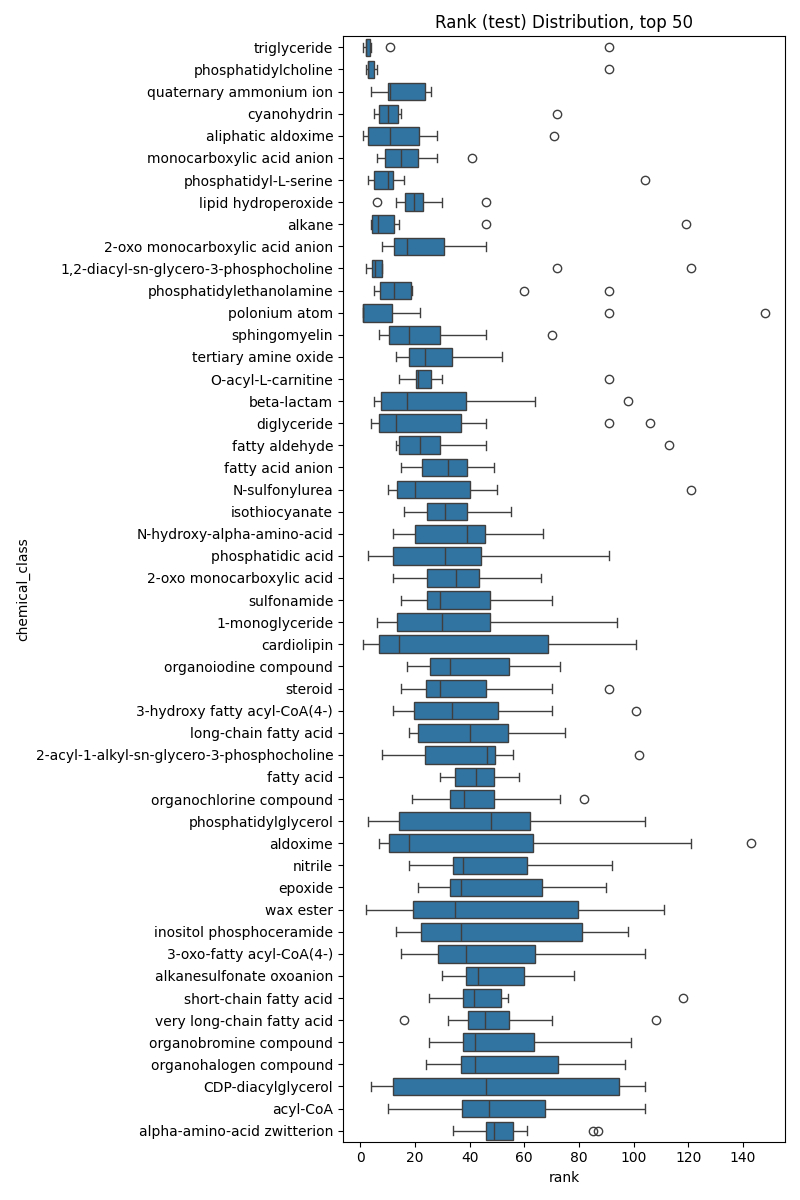

Supplement: Supplementary file 1 — Additional file 1 (Figure S1: Distribution of ranks for average top learnable classes. Each row is a chemical class, with the plot showing the distribution of relative rankings for each model. Classes such as trigylceride are ranked as among the best scoring broadly across all experiments, but in some outlier cases this ranked more poorly. Note that Figure 4 shows the most learnable classes for the ensemble, whereas this gives a picture of variation among models and hyperparameters.) [file 13321_2025_1092_MOESM1_ESM.jpg]

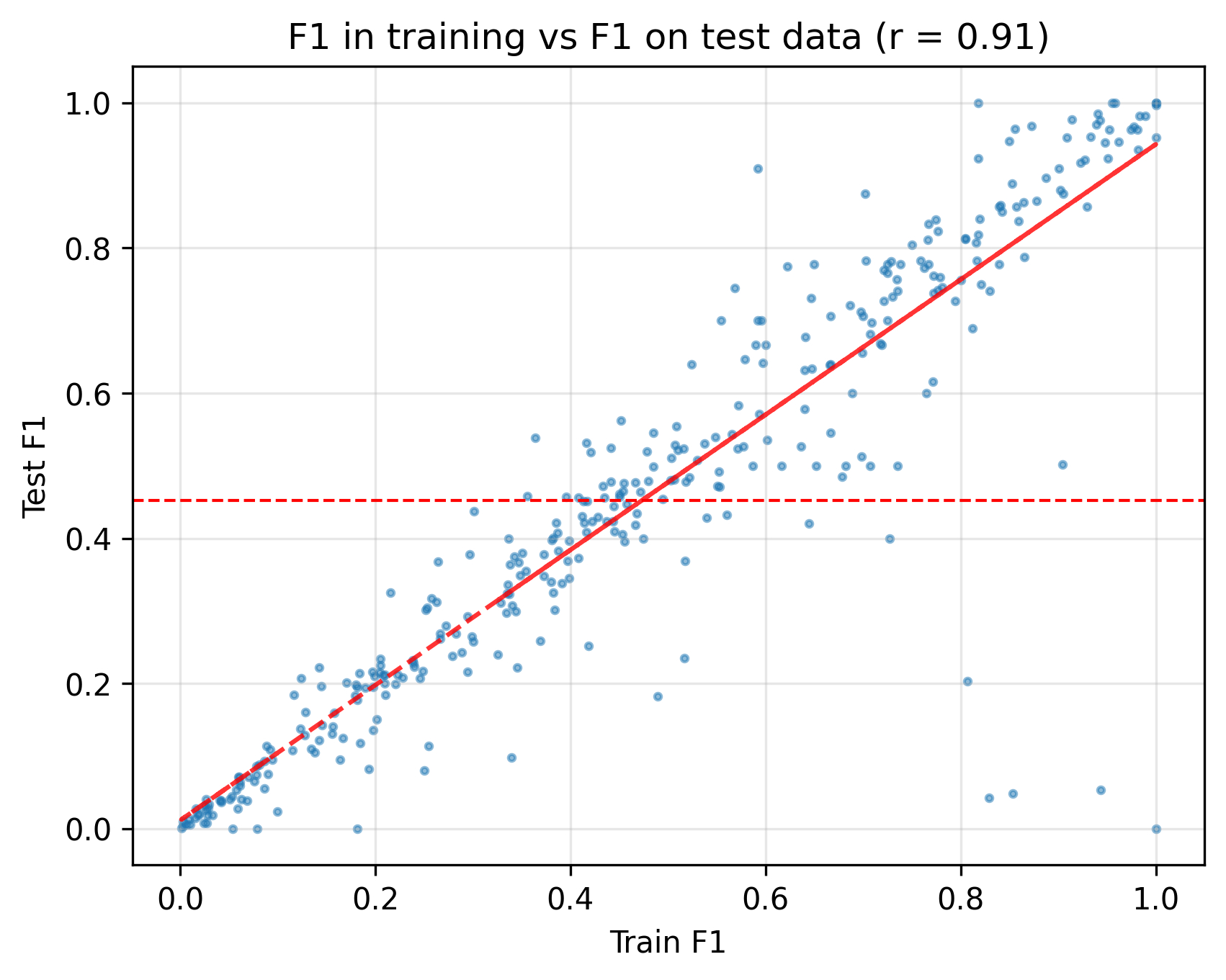

Supplement: Supplementary file 2 — Additional file 2 (Figure S2: Generalization from training to test F1 scores. Scatterplot of F1 score from training phase versus F1 score from held-out data in test phase, using ensemble model. There is a high correlation, with a broad distribution of scores. This indicates we can know in advance with some confidence which classifications will be reliable, i.e. more learnable classes predictably perform better.) [file 13321_2025_1092_MOESM2_ESM.jpg]

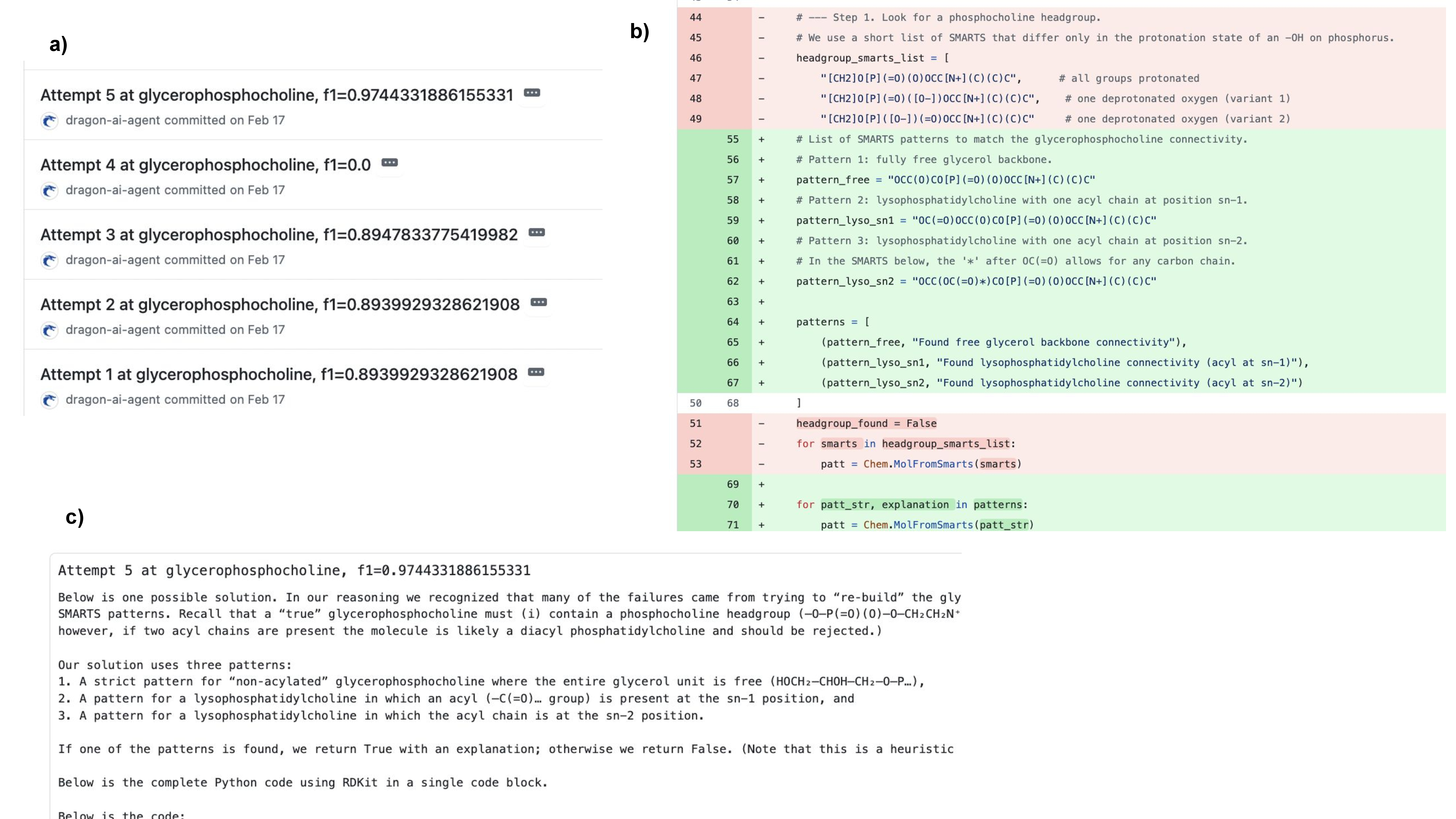

Supplement: Supplementary file 3 — Additional file 3 (Figure S3 Example of program convergence, for glycerophosphocholine. (a) GitHub log of attempts made by the agent to produce the best program (using only training examples), with progression from 0.89 to 0.97, with a temporary ‘blind alley’ exploration. (b) Example of one step program evolution, showing code difference between 4th and 5th attempt (c) agent’s rationale for the changes made to create the final (5th) attempt (stored as comments in GitHub).) [file 13321_2025_1092_MOESM3_ESM.jpg]

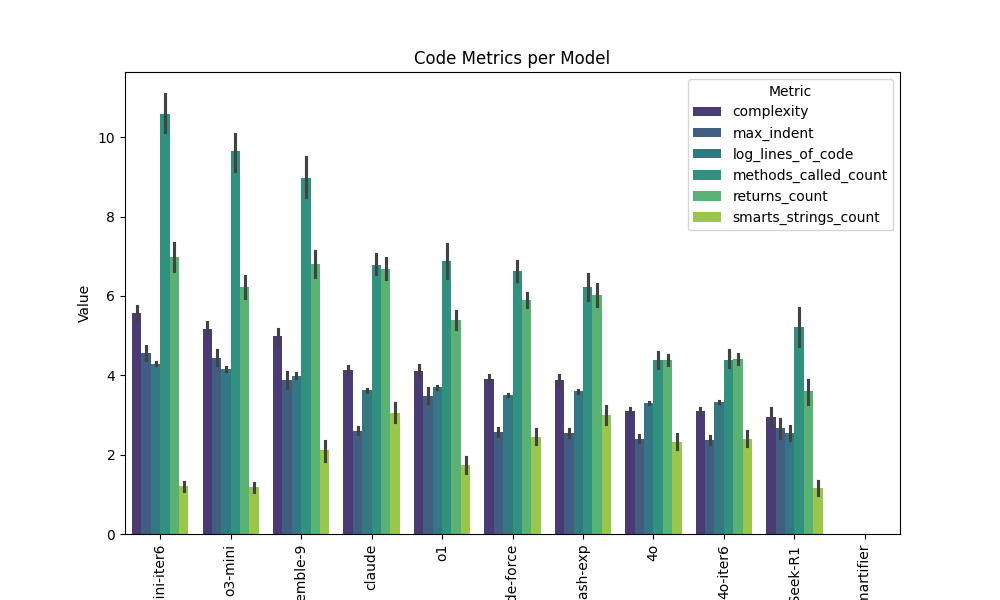

Supplement: Supplementary file 4 — Additional file 4 (Figure S4 Analysis of code properties of generated programs. Different metrics include total lines of code (log scale), number of distinct methods called, number of return points (as a proxy for different branch points in decision logic), the number of different SMARTS strings used. Error bars indicate 95% confidence. Overall o3 mini produced the most complex code, and gpt-4o (in all 3 configurations) was the most laconic, favoring shorter, less complex programs. Different models varied widely in how they used SMARTS strings, with Claude averaging the most, and o1 using them the least.) [file 13321_2025_1092_MOESM4_ESM.jpg]

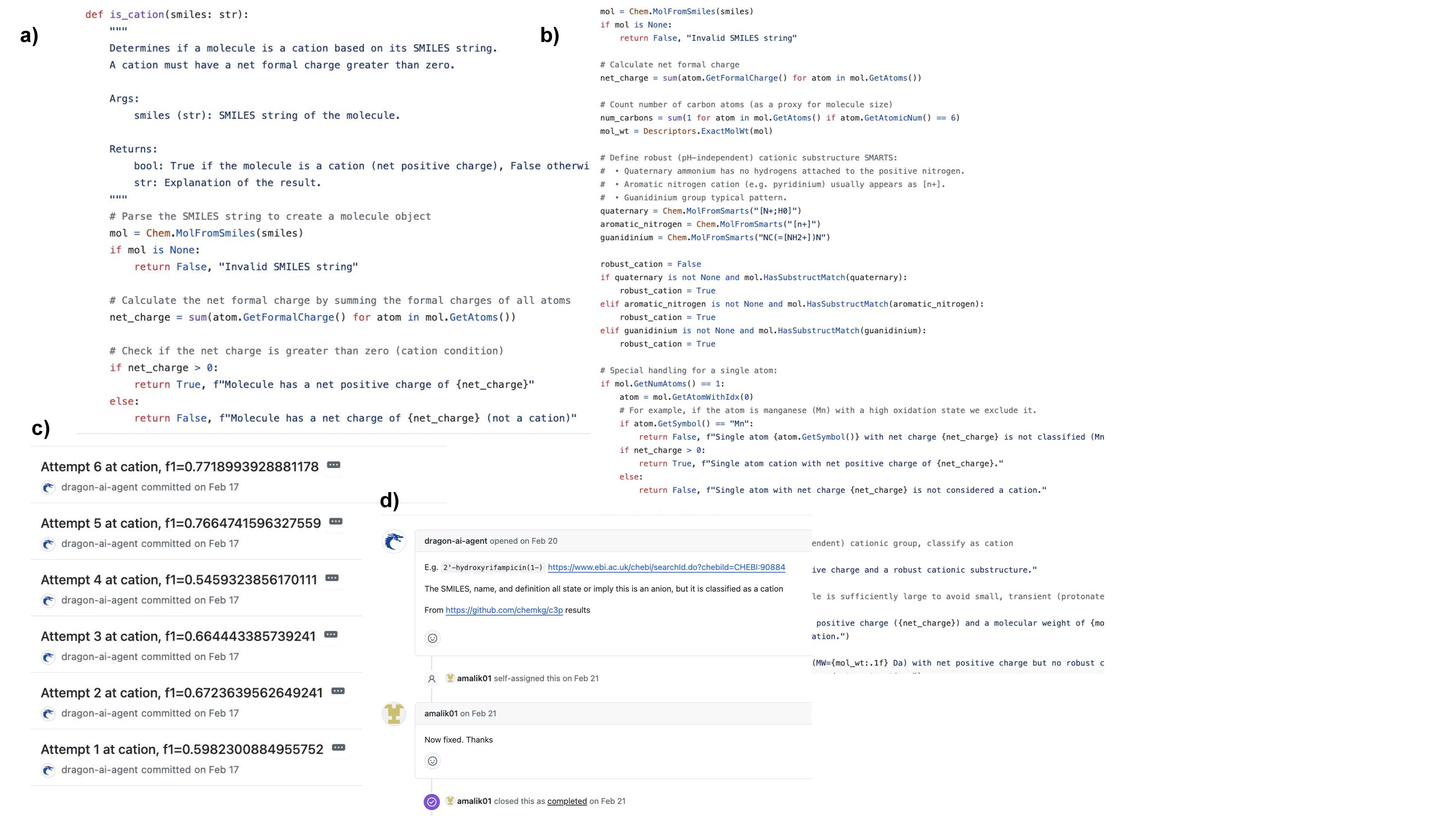

Supplement: Supplementary file 5 — Additional file 5 (Figure S5 Example of a case where errors in the training set cause learning to overfit on overly complex programs. (a) the initial first pass at a program is parsimonious and logically valid, yet this had F1=0.6. (b) the final program converged on after five subsequent attempts. This is considerably more complex, but it achieves a higher F1 score (c) evolution of F1 scores. (d) an example of an issue created in the ChEBI tracker highlighting the mistaken classification and resolution (in fact there were many other such misclassifications). Not shown is the thinking process for generating the final version of the program, repeated below: “Below is our “thinking‐out‐loud” summary before the code. In our last attempt the logic was very “binary” (simply net charge positive versus zero) so that many small, protonated species got classified as cations even though their positive charge is pH–dependent, while many zwitterionic molecules with a robust “cationic lipid” character (for example, many phosphatidylcholines) were missed. To improve the F1 score we (i) now distinguish “robust” cationic groups (i.e. quaternary ammonium, aromatic nitrogen cations, guanidinium groups) from protonated amines; (ii) we also require that—for molecules in the net–zero case—the compound must contain a phosphorus atom (as found in a phospholipid headgroup) and have a large carbon skeleton (we now require at least 30 carbon atoms) in order to call it a cation (this helps avoid mis‐calling small zwitterions as “cationic”); and (iii) for singly charged molecules whose positive charge comes from a protonated amine (i.e. not “robust”) we now impose a molecular‐weight threshold (approximately 200 Da) so that very small species (such as N–dimethylethanolamine, MW≈90) are not called cations. In addition, when a molecule is a single atom we accept it as a cation—except in one ad hoc case (for example, manganese in the unusual +7 oxidation state) which we explicitly exclude.”) [file 13321_2025_1092_MOESM5_ESM.jpg]
